# Pitfalls and potential of high-throughput plant phenotyping platforms

**DOI:** 10.3389/fpls.2023.1233794

**Published:** 2023-08-23

**Authors:** Hendrik Poorter, Grégoire M. Hummel, Kerstin A. Nagel, Fabio Fiorani, Philipp von Gillhaussen, Olivia Virnich, Ulrich Schurr, Johannes A. Postma, Rick van de Zedde, Anika Wiese-Klinkenberg

**Affiliations:** ^1^ Plant Sciences (IBG-2), Forschungszentrum Jülich GmbH, Jülich, Germany; ^2^ Department of Natural Sciences, Macquarie University, North Ryde, NSW, Australia; ^3^ Phenospex, Heerlen, Netherlands; ^4^ International Plant Phenotyping Network e.V. (IPPN), Jülich, Germany; ^5^ Plant Sciences Group, Wageningen University & Research, Wageningen, Netherlands; ^6^ Bioinformatics (IBG-4), Forschungszentrum Jülich GmbH, Jülich, Germany

**Keywords:** calibration curve, digital biomass, high-throughput plant phenotyping, leaf mass per area, sensors

## Abstract

Automated high-throughput plant phenotyping (HTPP) enables non-invasive, fast and standardized evaluations of a large number of plants for size, development, and certain physiological variables. Many research groups recognize the potential of HTPP and have made significant investments in HTPP infrastructure, or are considering doing so. To make optimal use of limited resources, it is important to plan and use these facilities prudently and to interpret the results carefully. Here we present a number of points that users should consider before purchasing, building or utilizing such equipment. They relate to (1) the financial and time investment for acquisition, operation, and maintenance, (2) the constraints associated with such machines in terms of flexibility and growth conditions, (3) the pros and cons of frequent non-destructive measurements, (4) the level of information provided by proxy traits, and (5) the utilization of calibration curves. Using data from an Arabidopsis experiment, we demonstrate how diurnal changes in leaf angle can impact plant size estimates from top-view cameras, causing deviations of more than 20% over the day. Growth analysis data from another rosette species showed that there was a curvilinear relationship between total and projected leaf area. Neglecting this curvilinearity resulted in linear calibration curves that, although having a high r^2^ (> 0.92), also exhibited large relative errors. Another important consideration we discussed is the frequency at which calibration curves need to be generated and whether different treatments, seasons, or genotypes require distinct calibration curves. In conclusion, HTPP systems have become a valuable addition to the toolbox of plant biologists, provided that these systems are tailored to the research questions of interest, and users are aware of both the possible pitfalls and potential involved.

## 1 Introduction

For decades, plant growth has been studied by taking non-destructive measurements such as plant height, along with destructive measurements such as plant dry mass. Typically, these assessments involved serial measurements over time, or comparisons of plants or plots at a ‘final’ harvest (Evans, 1972). Usually, 3-8 plants per treatment were harvested, or 2-30 genotypes compared, in a process that could easily take a full day or more for a single person. However, In the past 15 years, there has been a significant shift towards high-throughput plant phenotyping (HTPP). Fully automated systems now screen up to hundreds of genotypes and thousands of individual plants or field plots using non-destructive sensors, with the collected data automatically processed and stored for later use. (Furbank and Tester, 2011; Fiorani and Schurr, 2013; Tardieu et al., 2017; Lorence and Jimenez, 2022). In controlled conditions, automated phenotyping is often achieved by bringing individual plants to sensors (Yang et al., 2014; Al-Tamimi et al., 2016), or by moving sensors to or over the plants (Granier et al., 2006; Nagel et al., 2012). In the field, sensors are also brought to plants, either through mobile vehicles (White and Conley, 2013; Deery et al., 2014) or via drones or other aerial platforms that fly over field trials (Vargas et al., 2020; Roth et al., 2022). In all of these cases, automation has enabled a significant increase in the number of individual plants or plots that can be processed daily, often by an order of magnitude.

An important driver for the development of HTPP systems has been the rapid progress in the field of molecular biology. The extensive expansion and utilization of molecular tools at continuously decreasing costs have enabled thorough genotypic characterization of many plant species, cultivars, and genotypes. Phenotypic characterization, however, still lags behind, first because it is a time-consuming and often still manual measurement process, and second because plants of the same genotype can exhibit a range of different phenotypes, depending on environmental conditions (Furbank and Tester, 2011; Zavafer et al., 2023). This phenotyping bottleneck is particularly pronounced when studying traits that are controlled by multiple genes. In such cases, top-down approaches like Quantitative Trait Loci (QTL) analysis or Genome-Wide Association Studies (GWAS), are necessary to identify regions of the genome that are determining these traits (Gibson, 2018). However, conducting these analyses requires phenotyping hundreds of different genotypes, preferably all grown simultaneously in a common environment. Such analyses greatly benefit from the automation and standardization provided by HTPP systems (reviewed in Xiao et al., 2022).

Various technological advancements have facilitated the development of automated HTPP systems. A wide range of non-destructive sensors, including digital RGB cameras, hyperspectral, thermal, and fluorescence cameras, laser scanners, and LIDARs (Liu et al, 2020) enable repeated measurements of individual plants over time. This provides a higher resolution for capturing time-related phenotypic changes compared to experimental designs where new plants need to be harvested destructively for each time point (Poorter and Garnier, 1996; Walter et al., 2007). The use of automated gantries and transportation systems as well as drones allow us to minimize the distance between sensors and plants. Another significant factor is the increased computational power and improved algorithms that enable efficient image processing, with or without machine learning (Walter et al., 2015; Dobrescu et al., 2020).

As mentioned above, HTPP systems encompass a diverse range of approaches, all centered around non-destructive measurements. In the years ahead, more and more of these systems will be built to effectively screen a large number of plants for their size, growth trajectory, and other traits. However, like any complex equipment, the use of these platforms also presents challenges and limitations that may be overlooked by those who have not yet utilized them. Therefore, before investing in the purchase, construction, and utilization of these platforms, it is crucial to consider potential issues that may arise, and how they can be addressed. In the Results and Discussion section, we share insights gained from our experience in developing and deploying HTPP systems over the past 15 years. While our focus is primarily on systems operating in (semi-)controlled environments, several of the issues discussed will apply to field phenotyping as well.

## 2 Materials and methods

Part of the discussion that follows will address the relationship between non-destructive measurements, such as projected leaf area, and variables that require destructive analysis, including total leaf area, shoot biomass, and total plant biomass. We illustrate this part of the discussion with data from two experiments. In the first experiment, *Arabidopsis thaliana* (Col-0) plants were cultivated in a growth room using a rack equipped with neon tubes (Fluora, Osram, Munich). The plants were grown in soil (ED73, Einheitserde, Uetersen, Germany) in a cultivation tray with adjacent 80 ml cells. After incubating the seeds in the dark at 4°C, they germinated in the tray. The plants were then subjected to a 12-hour day length, a photosynthetic photon flux density (PPFD) of 40-50 μmol m^-2^ s^-1^, a day/night temperature of 23/20°C and a relative humidity (RH) of ca. 55%. Plants were watered from below when the top-soil was fully dry. Thirty-nine days after sowing, the plants were imaged six times during the day, with 2-hour intervals. Screening and image analysis were performed following Walter et al. (2007). The data from this experiment can be found in **Supplementary Data Sheet A1**.

The second experiment involved studying *Plantago major*, another rosette-forming species, throughout a significant part of its growth cycle and included a large number of replicates per harvest (n=12). The plants were grown hydroponically under two different CO_2_ concentrations (350 and 700 µmol mol^-1^), in growth chambers with a 12-hour day length, a PPFD of 230-270 μmol m^-2^ s^-1^, a day/night temperature of 20/18°C and a day/night RH of 60/90%. Plants from both treatments were monitored weekly over a 7-week growth period. Projected leaf area (PLA) was determined by capturing slides with an analogue camera placed at a height of 2 meters. The slides were projected, plant outlines traced on papers, and those were subsequently digitized semi-manually using a digitizer (model 9874a, Hewlett Packard, Stanford, CA, USA). Total leaf area per plant (TLA) was determined using a leaf area meter (LiCor 3100, Li-Cor Inc, Lincoln, NE, USA) equipped with a conveyor belt system. The determination of the total dry mass of shoots and roots was done manually, with plant size varying >300-fold throughout the course of the experiment. The data for this experiment is sourced from a previously published study by Poorter et al. (1988) and can be found in **Supplementary Data Sheet 2**.

Data were analyzed using R version 4.1 (R Core Team, 2022). Various calibration curves were established by employing the function lm, with or without a ln-transformation of the relevant variables, and with or without a quadratic term for the explanatory variable.

## 3 Results and discussion

**Figure 1:**
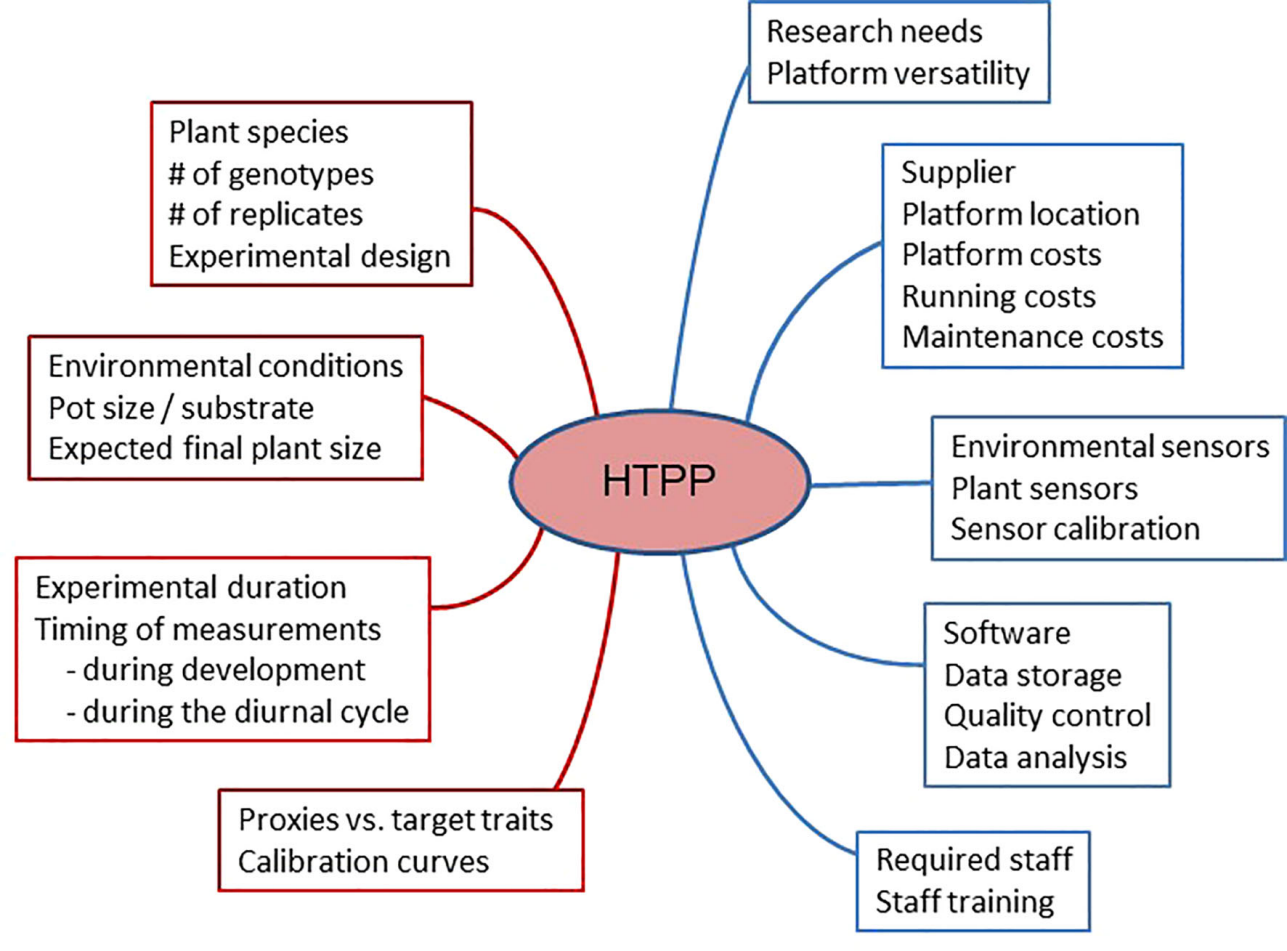
A schematic diagram indicating different aspects of high-throughput plant phenotyping (HTPP) systems that should be considered before purchasing such a system (in blue) and using it (in brown).

Before proceeding with the design and implementation of a high-throughput plant phenotyping (HTPP) system, it is essential to consider the following questions. **Figure 1** shows a schematic overview of the relevant aspects to be considered.

### 3.1 What is the specific research need?

HTPP systems are designed based on principles of automation and standardization. However, several critical aspects must be considered and discussed in order to effectively design and implement these systems. These include the required scale of the experiments, the selection of sensors that will be installed, the necessary software infrastructure, and the expected return on investment in terms of financial and human resources. These aspects can only be fruitfully discussed if guided by the question what purpose the system should serve and what goal(s) one would like to achieve through this platform. This is even more important in the case where multiple research groups with diverse interests are involved, as there is a risk that the intended platform becomes a compromise that in the end does not satisfy any.

### 3.2 What are the costs of investment and maintenance for a phenotyping platform?

HTPP systems involve a complex combination of logistics and technology, particularly when plants need to be moved to sensors. Such machines require the use of conveyor belts, gantries, or mobile robots to transport plants through growth chambers or glasshouses to an imaging station. At the imaging station, plants are generally subjected to controlled conditions, such as a consistent light spectrum and intensity, allowing for a consistent acquisition of images or other sensor-based data. Additionally, plants in pots or carriers can be automatically weighed and watered. Measurement equipment must be linked to large-scale data storage facilities and databases, in order to store and retrieve the collected measurement information and other metadata. Clearly, expert knowledge of automation, logistics, error control, fine mechanics, non-destructive sensing, image analysis, and database management is required to build and operate these systems properly. Companies specializing in these different fields offer to construct customized machines on-site. The costs of these systems can be substantial. Prices typically range from approximately €60,000 to €120,000 for small systems with a lower degree of automation and throughput, €350,000 – €500,000 for fully automated sensor-to-plant or plant-to-sensor systems, and up to €3,000,000 for high-end systems with extensive optimization and automation of the workflow. The costs vary depending on factors such as the types and number of sensors used, system size, quality of the product and service provided by the supplier. In addition, these custom-built facilities require expert maintenance, including sensor calibration, replacement of worn-out parts and preventive check-ups. Yearly servicing contracts often amount to 5-10% of the acquisition price and are necessary for the duration of system usage.

Given the above considerations, certain laboratories opt to construct HTPP systems themselves, either utilizing off-the-shelf products or with the support of robotics companies (Bagley et al., 2020). Building customized platforms can allow for increased flexibility (as discussed in section 3.4) and better fit the specific requirements of the involved labs. However, it is important to acknowledge that the path to a fully functional machine is challenging and time-consuming. While the direct hardware costs may be lower than those of commercial HTPP systems, the substantial human resources required for the development and maintenance of such automated systems should not be underestimated (Reynolds et al., 2019).

There are two additional points to consider regarding personnel investment. Firstly, it is advisable to involve expert users, project managers, and engineers throughout the planning and building phase. This ensures that any necessary compromises that almost inevitably have to be made during the whole process do not strongly constrain the desired goals of the end-users. Secondly, operating these systems can be complex, particularly when troubleshooting errors, as there are numerous variables to consider within the platform software, platform mechanics and network communications. Therefore, it is recommended to have an operational team led by an expert who is responsible for the platform. This lead expert can receive training from the supplier or the constructors, and subsequently train all other individuals who will be utilizing this platform. Clearly, such a person requires both technological as well as plant biological expertise to effectively manage and operate the HTPP system.

### 3.3 What other logistical and infrastructural adjustments are necessary?

An HTPP platform allows researchers to significantly scale up their experiments, often by an order of magnitude. This implies that also an order of magnitude more plants are processed, more containers have to be filled, more consumables are used, more electricity and irrigation are required, and additional cleaning has to be done at the end of the experiment. While these aspects are not part of the phenotyping platform *per se*, they contribute to a significant workload. As a result, there is often a need for subsequent adjustments to the workflow’s ergonomics, such as automating tasks like pot filling, seed sowing (Jahnke et al., 2016) or plant transplantation. Together with the sampling for further physiological characterization and disinfection or disposal of used components, they bring about additional planning and investments on top of those for the platform itself.

Another aspect to consider is the data infrastructure. Given the massive amount of data generated by HTPP systems, effective storage and accessibility of data for both short-term and long-term use are essential. Such utilization of data requires a good documentation of data and metadata, as described in the ‘Minimal Information About Plant Phenotyping Experiments’ (MIAPPE) guidelines (Krajewski et al., 2015; Papoutsoglou et al., 2020). Also, data and metadata should be stored in accordance with the FAIR principle (findable, accessible, interoperable, and reusable; Wilkinson et al., 2016) as in GnpIS (Pommier et al., 2019) and as currently established in research data management infrastructures like DataPLANT (www.nfdi4plants.de).

Remote support by the different suppliers requires a safe, secure, and reliable access policy. Moreover, in the analysis of data from such platforms, there is a growing emphasis on integrating genotypic and phenotypic data, as well as incorporating detailed environmental characterization of the experiments. Bringing all these data together often requires a considerable *a-posteriori* effort from trained personnel, which also needs to be budgeted in advance to make full use of the potential of these HTPP systems (Reynolds et al., 2019).

### 3.4 How flexible can or should the system be?

Automation can replace a considerable amount of tedious repetitive manual labor, especially when routine operations are customized to the research question and species of interest. However, this increased automation often comes at the cost of reduced flexibility. For instance, a system that is perfectly fit for small rosette plants like *Arabidopsis thaliana*, may not be as suitable for a large species with different architecture, such as *Zea mays*. Increased flexibility of the system can be achieved by planning for modular components that can be interchanged depending on the prevailing research question. However, increased flexibility to accommodate the needs of a variety of researchers also implies that the system becomes increasingly complicated, with a higher likelihood of failures and more efforts to solve these problems. A ‘jack-of-all-trades’ system will hardly provide the same level of detail and raw data resolution across the entire range of plant sizes and architectures as a dedicated high-resolution system designed specifically for either small or big plants, due to limitations imposed by the optical properties of the sensor and system layout. In those cases, a potential solution could be to have two smaller and targeted systems, rather than relying on a one-size-fits-all solution.

Another aspect of flexibility is the expected lifespan of system components. Technological advancements occur rapidly, and new sensors, for example, will generally be more powerful and informative than their predecessors. However, owners of commercially-acquired phenotyping systems often face problems in that the software to run the whole system is proprietary to the company, and therefore not accessible for further development. In such cases, it is complicated or impossible to integrate new sensors or apply other modifications to existing HTPP systems. New buyers are advised to discuss with their suppliers what support they can get in that respect. Self-builders are suggested to set up their software interfaces as flexible as possible, to easier adjust their system when new sensors or updates become available.

### 3.5 What constraints does the system impose on growth conditions?

Plants are strongly influenced by their environment and different genotypes or species may show varying degrees of genotype x environment interactions for many of their phenotypic traits. This becomes particularly critical, because in most (semi-)controlled environments we impose abiotic conditions that significantly deviate from the natural conditions plants experience outdoors (Poorter et al., 2016; Chiang et al., 2020). Considerations about the location of the HTPP system (growth chamber, glasshouse, or field) and the range of environmental conditions provided to the plants are therefore an integral part of the design process.

Compared to traditional experiments, the use of high-throughput phenotyping systems often introduces additional constraints, that can impact the growth environment of the plants and, consequently, the outcomes of experiments. For instance, in a typical plant-to-sensor system, the combined weight of plants plus pots is limited by the strength of the conveyor belt used, as well as the scales used for gravimetric measurements. This limitation results in experiments being confined to plants in relatively small pots with substrates of low specific mass, which clearly affects plant growth and experimental outcomes (Passioura, 2002; Poorter et al., 2012). Transportation may also have other consequences. Tall plants, such as *Zea mays*, may topple over if the conveyor belts move too quickly. During transport, leaves of sensitive species (e.g. *Brassica rapa*, *Hordeum vulgare*) can get damaged, causing them to droop downwards along the pot. To mitigate damage, researchers may choose to place suitable support structures next to or around each plant, except in cases where wilting would be a phenotypic trait of interest. Apart from direct damage, one would also expect thigmo-morphogenetic responses to occur as a reaction to the mechanical perturbations during transport of plants, such as thicker and shorter stems (Anten et al., 2005). However, plant height was not negatively affected in the experiment of Brien et al. (2013), and neither was shoot biomass or leaf area in various experiments (**Table 1**).

**Table 1:** Effect of plant-to-sensor transport on shoot biomass, as based on various experiments carried out in glasshouses.

Reference	Mode of transport	Species	Measured variable	Size ratio of shoots (moving vs. non-moving plants)	P
Nagel et al. (2020)	pneumatically	*Arabidopsis thaliana*	LA	0.96	ns
F. Fiorani & N. Körber (unpubl.)	gantry system	*Hordeum vulgare*	FM	0.97	ns
F. Fiorani & N. Körber (unpubl.)	gantry system	*Brassica napus*	FM	1.02	ns
Brien et al. (2013)	conveyor belt	*Triticum aestivum*	FM	1.03	ns
Junker et al. (2015)	conveyor belt	*Arabidopsis thaliana*	DM	1.08	*

LA, Leaf area; FM, Fresh mass of the shoot; DM, Dry mass of the shoot. Statistical significance: *, P < 0.05; ns, non-significant. Included are the mode of transport of the plants, the species investigated, variable measured, the size ratio of shoots of plants that were moved relative to control plants that were not moved, and the statistical significance of these differences in plant mass. The experiments are ranked based on the observed effect size.

**Figure 2:**
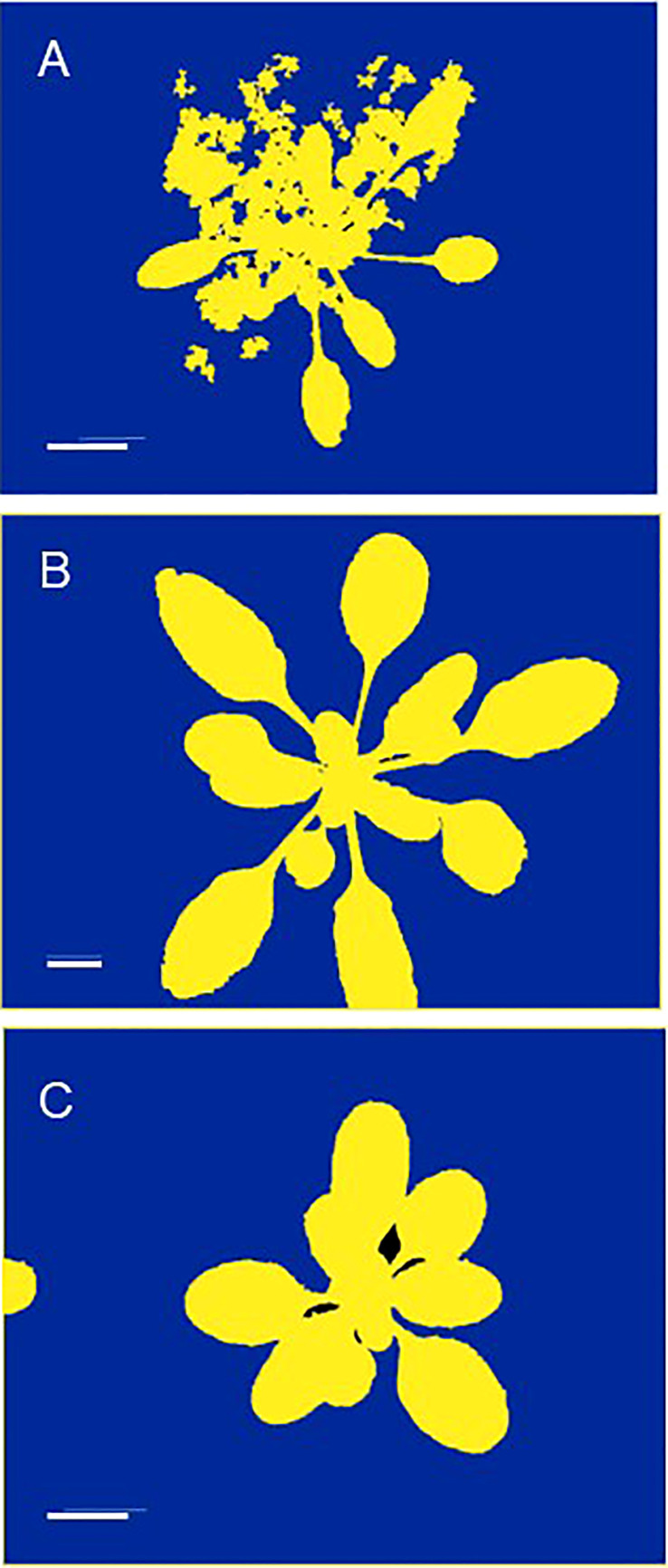
Examples where unsupervised automated high-throughput phenotyping may lead to incorrect results. **(A)** Algal growth resulting in a mask that is too broad and yields an overestimation of projected leaf area (PLA). **(B)** A plant grown out of the image acquisition area, resulting in an underestimation of PLA. **(C)** A neighboring plant growing into the image acquisition area, resulting in an overestimation of PLA. All pictures depict *Arabidopsis thaliana* plants and are masked images, used for measuring PLA by counting the number of green pixels. These images are for illustrative purposes only. The scale indicates a length of 1 cm.

Fixed distances between cameras/sensors and plants may restrict the range of plant sizes that can be investigated, thereby limiting the developmental stages that can be studied in such HTPP systems to young vegetative plants. A last example pertains to the watering of the plants. Well-watered containers, especially with peat substrate, often show algal growth on the soil surface, which hampers the non-destructive derivation of plant size through image analysis (**Figure 2A**). To avoid this complication, researchers may opt to cover the top of the pots with a plastic sheet of contrasting color (Junker et al., 2015). Alternatively, for small rosette species like *Arabidopsis*, reducing the volume and/or frequency of watering can keep the topsoil dry for a longer duration. Although this suppresses algal growth, it also has unknown consequences for the growth rate and phenotype of the plants. It is recommended to consider *a-priori* whether the additional constraints imposed by an HTPP system are acceptable within the scope of the research question.

### 3.6 When and how often should the plants be measured?

Once an operational HTPP system is in place, attention can shift to the performance of experiments. Given their typical large scale, careful consideration of experimental design is essential. For a comprehensive discussion on this topic, the reader is referred to Thompson et al. (2022). One notable advantage of high-throughput phenotyping systems is that individual plants (or microplots) can be measured frequently and non-destructively. This allows for the repeated measurements of the same plants, enabling the tracking of their growth and development over time. Such analyses can yield valuable insights, with a good example discussed in **Box 1**. However, in cases where the research question focuses on identifying the best-performing genotype at the end of the experiment, repeated measurements may be unnecessary. In those cases, researchers could also opt for one final destructive harvest, which might be more simple, cheaper, and more informative, as also illustrated in **Box 1**. A lower measurement frequency may also be advantageous if the measurements have the potential to interfere with plant growth. This is particularly relevant when plants are taken out of their growth environment for longer-duration measurements, such as magnetic resonance imaging (MRI) or computer tomography (CT) scans. A higher measurement frequency then provides better insight into plant development, but may also have a stronger negative effect on plant growth.

Box 1 **Example of the trade-off between measuring with higher frequency or higher precision.**

**Figure 3:**
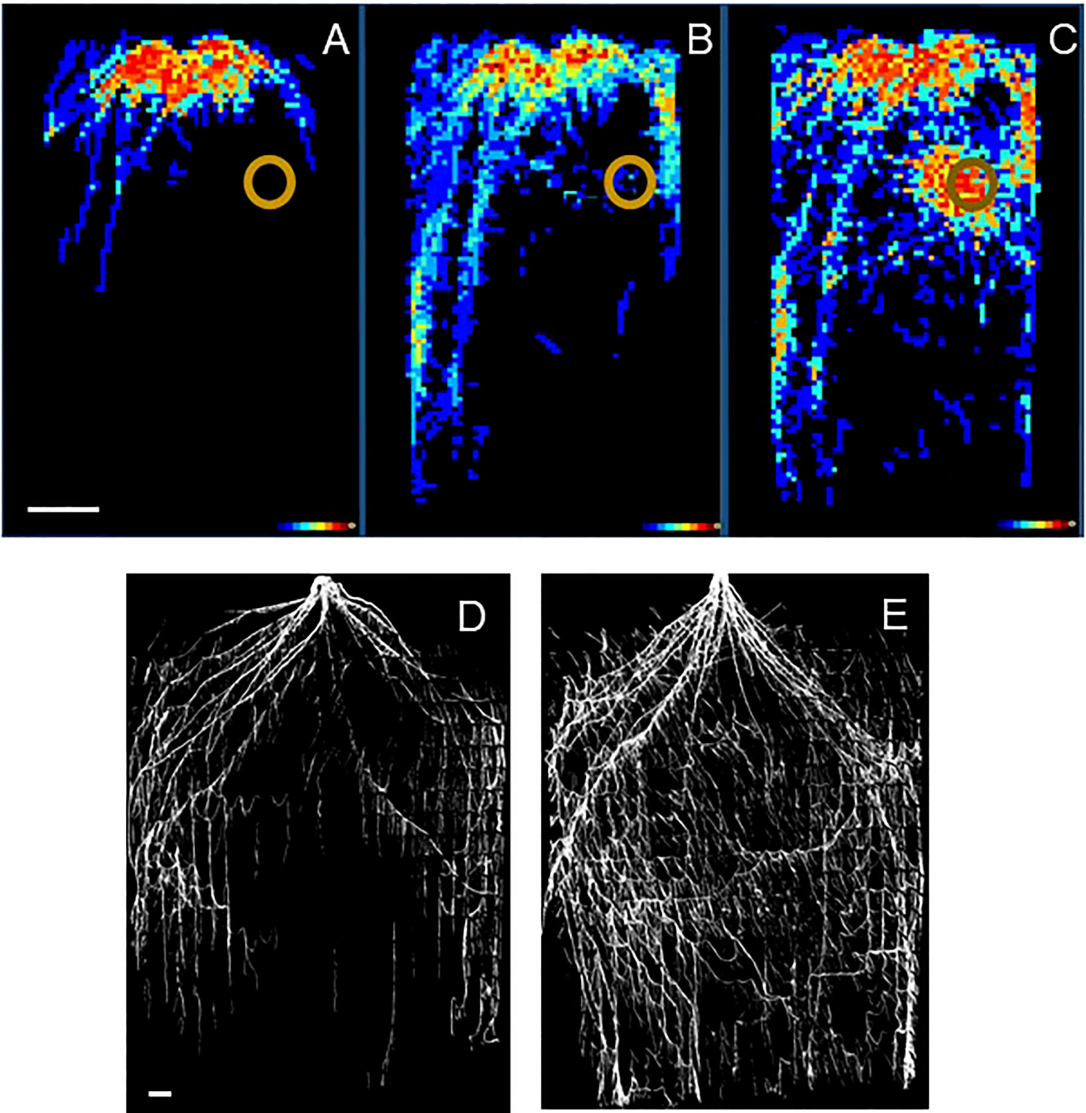
**(A–C)** Root distribution of *Sida hermaphrodita* plants in rhizotron boxes with a localized depot of digestate, indicated by the brown circle. **(D)** Root distribution in a rhizobox at the end of an experiment after the soil has been removed. Figures **(A–C)** are adapted from Nabel et al. (2018) and show in false colors ranging from dark blue to bright red the number of rhizotrons (out of 10 in total) where roots were observed for each x-y location in the rhizotron. **(A)** 30 days, **(B)** 60 days and **(C)** 90 days after the start of the experiment. Figures **(D, E)** show the root systems of two *Sorghum bicolor* genotypes at the 12th-leaf stage (ca. 6-8 weeks after germination) in an experiment similar to that described in Singh et al. (2010). The white bars indicate a length of 10 cm. Picture credits Figures **(A–C)**: Moritz Nabel, Forschungszentrum Jülich, Germany. **(D, E)**: Vijaya Singh, University of Queensland, Australia.

Here we provide two examples of experiments focusing on root distribution. The first one followed root development non-destructively over time (Nabel et al., 2018). Seedlings of a woody shrub (*Sida hermaphrodita*) were grown for 90 days in rhizotrons of 36 x 75 x 2.6 cm in size, where one side consists of transparent acrylic glass. The researchers placed digestate, which is a residue remaining after anaerobic digestion of biomass to methane, at a specific location in the rhizobox. This location is indicated by the brown circles in **Figures 3A–C**. Root growth of the part of the root system close to the transparent side of the rhizobox was monitored by regularly capturing images (Nagel et al., 2012). These images showed that plants strongly avoided the digestate patch in the first 60 days of the experiment (**Figures 3A, B**). However, roots strongly proliferated into this patch of nutrients later in time (**Figure 3C**). In this case, the timing proved to be an essential aspect of how these plants reacted to the treatment. Although these analyses of root distribution are still challenging for computers and often need human supervision, the effort in this case proved worthwhile for understanding the timing of root responses.

An alternative approach was followed by Singh et al. (2010; **Figure 3D**). They grew two *Sorghum* genotypes in rhizotrons measuring 120 x 240 x 10 cm. When it comes to selecting, for example, the best-performing genotypes of a panel of genotypes, an evaluation at the end of the experiment could be as informative as a complete analysis over time. If this is the case, researchers may also opt for alternative and cheaper set-ups, such as rhizoboxes that are not integrated into automated HTPP systems. Those rhizoboxes could have larger dimensions and allow a wider range of root substrates. By pushing a pinboard into one of the sides before washing away the soil with water, the distribution of a *whole* root system can be characterized, rather than only those roots close to the acrylic glass (Singh et al., 2010; **Figures 3D, E**).

The dilemma faced by researchers in these cases is whether it is more informative to have estimated data over the course of the experiment for only a portion of the root system, as provided by automated rhizotron systems, or to have more precise data capturing the entire root system but only at the end of the experiment.

**Figure 4:**
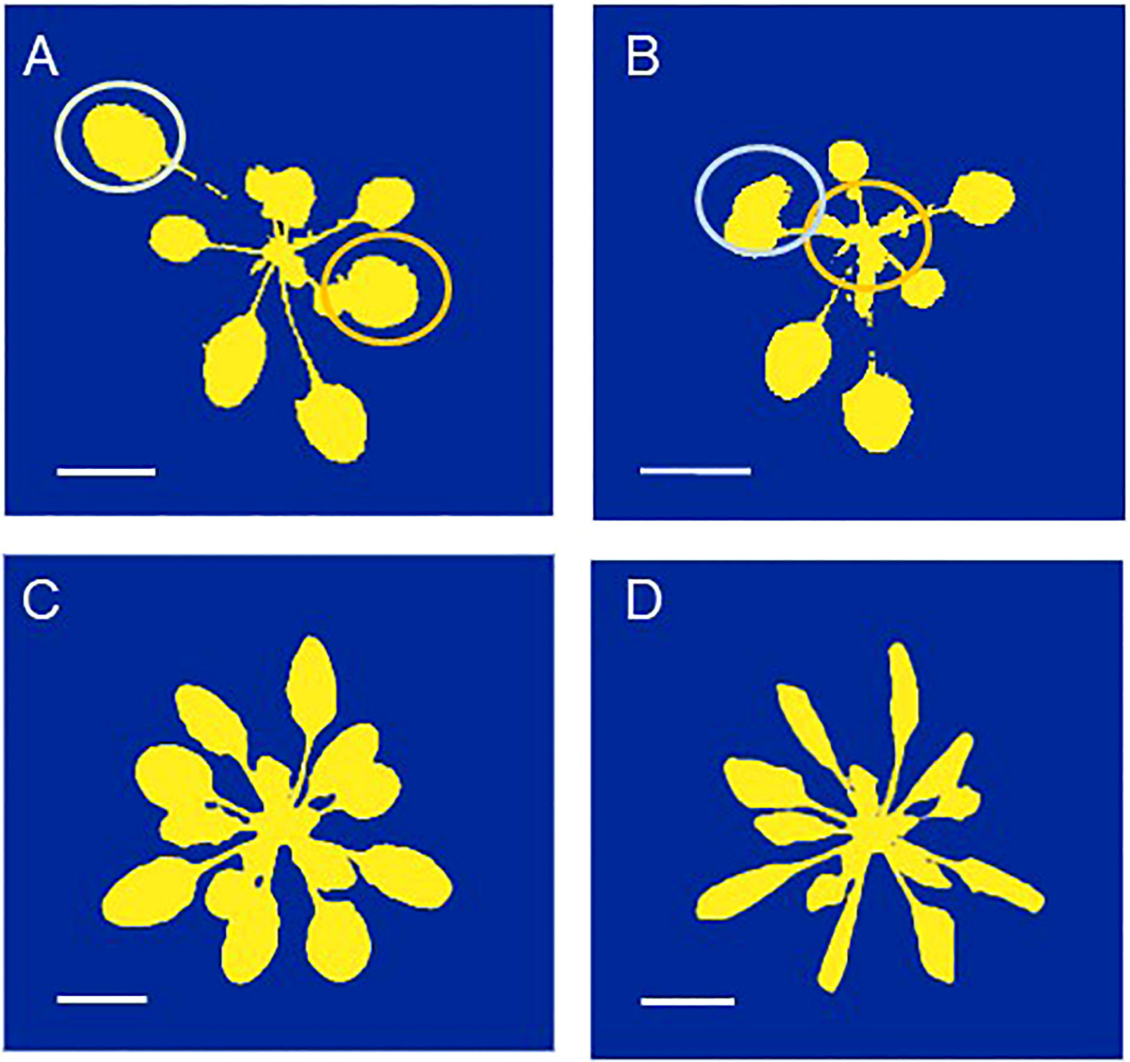
**(A, B)** The same *Arabidopsis thaliana* plant photographed **(A)** in the morning, with a low leaf angle and **(B)** at the end of the day with a much higher leaf angle. The yellow and orange circles indicate the positions of the youngest full-grown leaves which exhibit the largest change in leaf angle. **(C, D)**. The same plant photographed **(C)** before and **(D)** after the onset of water stress, which resulted in leaf wilting. These images are for illustrational purposes only. The scale indicates a length of 1 cm.

**Figure 5:**
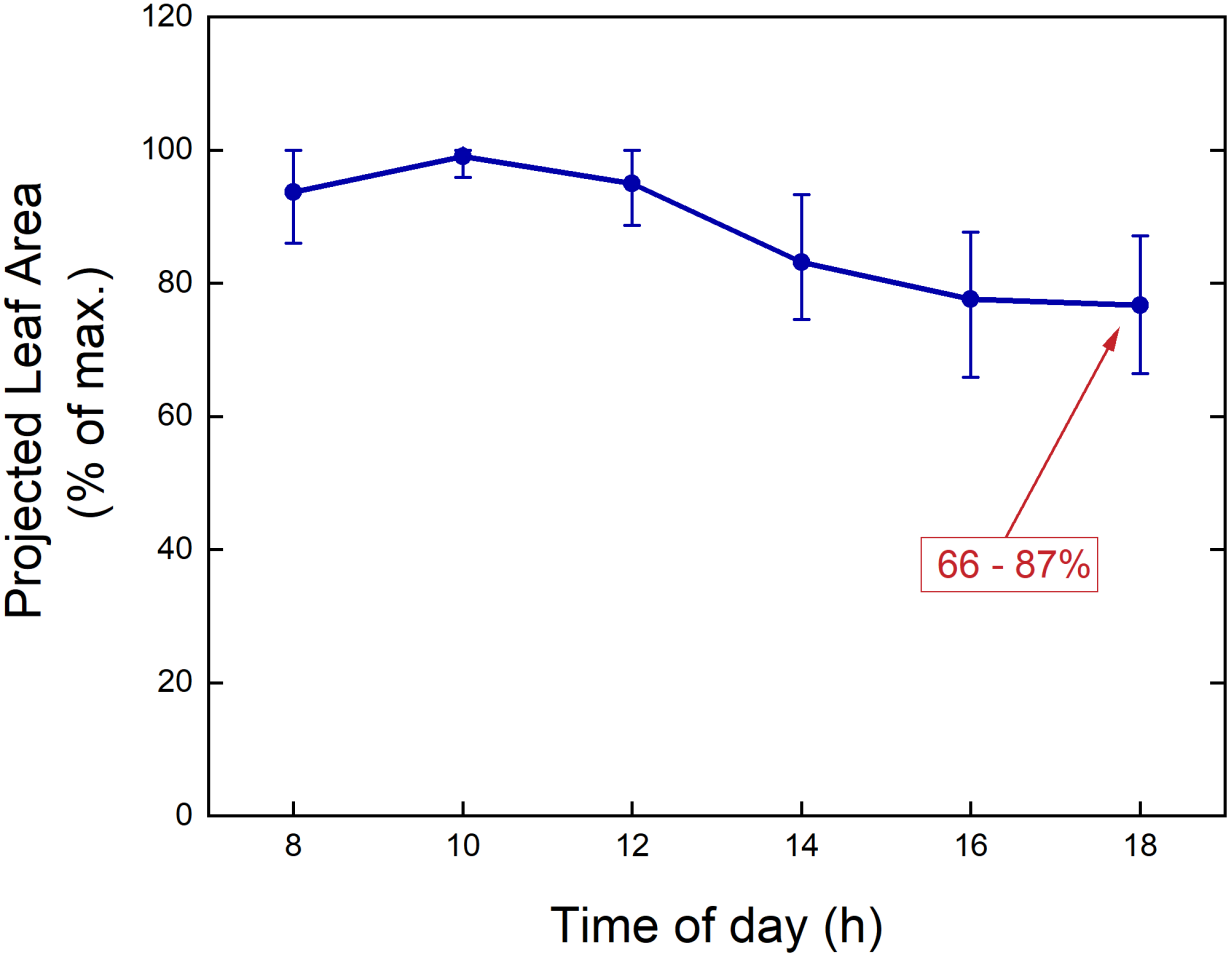
Measured Projected Leaf Area (PLA) over a day for *Arabidopsis thaliana* plants grown in a growth chamber. The values for each plant are normalized to the maximum value measured during the diurnal part of the day-night cycle. The dots indicate mean values, and the ‘error bars’ represent the 5% and 95% percentiles (n = 44).

A second point to consider is the time of day when plants will be measured. When using a drone to fly over an experimental field, it can capture images of many microplots simultaneously. Acquiring images or other data for these plots using sensors mounted on vehicles will take longer. Depending on the type of measurements taken, it may require minutes or more per microplot. With plant-to-sensor systems within a glasshouse, where it takes minutes to move a plant through the imaging station and measure its characteristics, phenotyping all plants in one experiment could take a full day. During that period, large changes in environmental variables, such as light intensity and temperature, may occur, likely resulting in large variation in physiological variables such as stomatal conductance and photosynthesis as well. One morphological trait that can exhibit considerable diurnal variation is leaf angle (Rosa and Forseth, 1996), which has obvious implications for the projected leaf area (PLA; Dobrescu et al., 2017) as used in many HTPP systems. For example, during the diurnal part of the diel cycle, *Arabidopsis* plants may *increase* their leaf area and biomass by 20% (Wiese et al., 2007). However, in a similar experiment, PLA values were found to *decrease* by 18-35% during the light period (**Figures 4A, B**, **5**), due to upward movements of leaves and petioles. Consequently, when conducting consecutive 2D measurements of plants day after day, it is important to measure them at the same time of day, to avoid bias caused by the diurnal rhythm of leaf movements. Moreover, different genotypes or treatments should be blocked into the same time window, to ensure that no confounding effects occur. One fast alternative approach is to perform a 3D laser scan (Dornbusch et al., 2012). Other options include using multiple imaging stations, or employing a gantry system where sensors are brought to the plants, enabling parallel measurements of many plants in short time.

In some cases, the research question requires a high frequency of measurements on the same plants. Examples are physiological responses of plants following the application of a compound, exposure to a pathogen, or exposure to abiotic stress (Jansen et al., 2009; Mahlein et al., 2019). In some species, capturing images at higher frequencies and analyzing them in almost real-time can be used to detect the onset of potentially undesired drought stress during the experiment (**Figures 4C, D**; Eberius and Lima-Guerra, 2009; Janni et al., 2019).

### 3.7 How adequate is the quality control and data handling?

The amount of information collected from a single experiment can be substantial, especially when imaging of any kind is involved. The question is how well we, as experimenters, can handle this vast quantity of data (Eberius and Lima-Guerra, 2009; Tardieu et al., 2017). Based on our own experience, we have observed several issues that can arise during an experiment. Sensors, especially environmental sensors that are deployed throughout the year, may have not been calibrated for a long time or show failures of various kinds during the experimental period. With larger amounts of plants, problems might more easily go undetected. For example, some plants could topple over or do not receive adequate watering. In image analysis systems, leaves from neighboring plants might appear in pictures taken, photos might not cover the full plant, or masking might not function properly (**Figures 2B, C**). These errors are easily noticed if they occur frequently, but in the midst of hundreds of plants, thousands of pictures, and tens of thousands of other collected data points, such errors can easily go unnoticed. Thorough data inspection and double-checking for mistakes are therefore crucial, but can be cumbersome without the assistance of digital tools. Dedicated software programs for data visualization and targeted image retrieval, such as Azure, iRods, PHIS, Fairdom, Zegami, or similar solutions, enable fast selection of images of specific plants over time, aiding in the identification of potential outlier data. Automated quality control procedures should routinely flag instances where parts of leaves are outside the picture, or leaves of neighboring plants are distorting the results. Graphical analysis of data distribution, time courses, or dose-response curves can provide insights into potential issues with specific plants or entire groups of plants (Xu et al., 2015). This is particularly useful in ongoing experiments, when possible problems can be detected and solved by data analysis at an early stage. Real-time reporting, along with easy and user-friendly (remote) access to visualizations and resulting analyses, facilitates early detection and problem-solving.

### 3.8 How informative are the selected proxies for the actual variables of interest?

For decades, scientists have relied on spectrophotometric measurements to asses enzyme activity, wet digestion or pyrolysis for leaf nitrogen determination, infra-red gas analyzers to determine photosynthesis, and manual harvesting to measure plant biomass. However, these conventional measurements all require significant manual effort, and are therefore not suitable for high-throughput phenotyping. Efforts have been made to automate such measurements (e.g. Gibon et al., 2004; Gomez et al., 2010), but challenges remain in automating processes such as grinding and weighing, particularly under low-temperature conditions to prevent chemical degradation (Hall et al., 2022). In search of alternatives, scientists have explored measurements that are easier to perform, yet still provide valuable information. For example, leaf nitrogen content can be estimated non-destructively using multispectral analysis (Ye et al., 2020), photosynthesis can be assessed through fluorescence, and biomass by counting green pixels in plant images.

Are these proxies informative enough? The chlorophyll fluorescence parameter (F_v_/F_m_), for instance, is often measured, but in many cases variation in F_v_/F_m_ does not reflect variation in the actual rate of photosynthesis (Poorter et al., 2019). The electron transport rate offers a better approximation, but still does not capture the true rate of C-fixation (Kalaji et al., 2014). Similarly, estimating digital biomass based on the number of green pixels in an image can provide an indication of plant size, but does not give the actual biomass, or information on biomass allocation to leaves, stems, and roots. This lack of information hampers comparisons across experiments, platforms, and the published literature. However, advancements in machine learning techniques now enable the segmentation of 2D or 3D images into leaves and stems (Golbach et al., 2016; Jin et al., 2019; Shi et al., 2019), so far only for smaller and/or specific species. Moreover, combined shoot and root phenotyping is feasible in rhizotron or agar-based platforms (Nagel et al., 2012; Nagel et al., 2020). These developments hold the potential to extract more comprehensive information from these photographs or raw sensor data.

In all cases, users must maintain a critical approach to their data, particularly when changes in plant morphology occur. A clear example is observed in drought-stressed plants where a loss in turgor can result in leaf rolling or wilting, leading to a noticeable decrease in projected leaf area (PLA; **Figures 4C, D**), while actual dry biomass is little affected. Even when the variable of interest is directly provided by sensors, it is wise to verify the definitions used. For one researcher, plant height may be the distance from the shoot basis to the highest plant part, for another it could be the distance from the basis to the apical meristem. In some non-destructive systems, a more statistical approach is taken, where this variable is defined as the average value of the pixels (or voxels) from the 80^th^ till the 90^th^ percentile with respect to height (Kjaer and Ottosen, 2015). Ideally, these definitions are included in the meta-data provided by the HTPP system.

### 3.9 Are non-destructive measurements sufficient?

The phenotype of plants is multifaceted, composed of hundreds of variables related to anatomy, morphology, chemical composition, carbon and water economy, growth, as well as reproduction (Lambers and Oliveira, 2019; Poorter et al., 2022; Zafaver et al., 2023). While some of these variables can be estimated non-destructively, the majority of plant traits require destructive sampling or harvesting. Consequently, HTPP systems, which are primarily non-destructive by nature, can only cover a subset of the phenotypic traits that researchers would ideally like to measure. However, by bringing sensors to the plant, additional measurements may become feasible. For example, continuous monitoring of transpiration over a plant’s lifespan in real time can be achieved by placing plants on a balance (Tardieu et al., 2017; Dalal et al., 2020). Nevertheless, many other traits can only be measured through destructive sampling or harvesting, which necessitates additional planning and manpower.

A highly promising advancement is the development of robots capable of approaching a plant and taking a leaf punch from a specific leaf blade (Alenyà et al., 2013; Foix et al., 2018). By promptly storing these samples in liquid nitrogen, a broad array of relevant biochemical analyses can be conducted, including the assessment of key metabolites and RNA expression levels (Hall et al., 2022).

### 3.10 What calibration curve is required?

**Figure 6:**
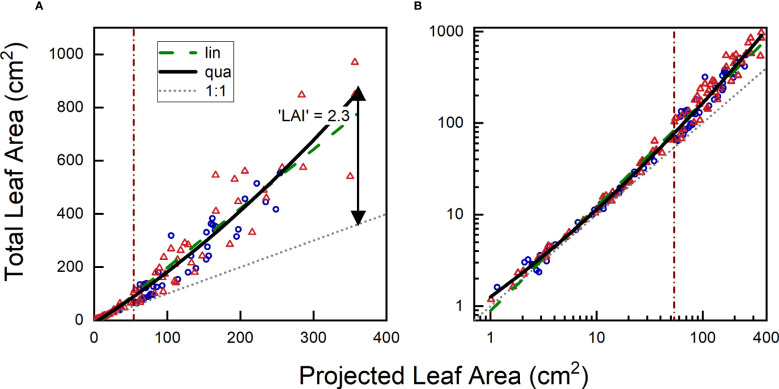
Relationship between projected leaf area and total leaf area of *Plantago major* plants harvested over an 8-week period and plotted on **(A)** linear or **(B)** logarithmic scales. Blue circles represent plants grown at low [CO_2_] (350 µL L^-1^), and orange triangles representplants grown at high [CO_2_] (700 µL L^-1^). The grey dotted line represents the 1:1 relationship between TLA (Total Leaf Area) and PLA (Projected Leaf Area), the green dashed line represents the linear fit through the data, and the black continuous line shows the quadratic fit. The vertical brown line indicates where the divide is between the 50% smaller and 50% larger plants, based on PLA.

In certain cases, well-calibrated phenotyping equipment can directly provide data on physiologically relevant variables of interest. For instance, measurements such as leaf temperature or F_v_/F_m_ yield output that is readily biologically interpretable and can be easily related to published work in the literature. In other cases, however, additional calibration is required to transform acquired data (e.g., number of green pixels) into biological meaningful variables (e.g., shoot dry mass). In such cases, a common procedure involves periodic measurements of a subset of plants, initially through non-destructive imaging, and subsequently destructively by determining leaf area, shoot dry mass or other traits of interest. An example is shown in **Figure 6**, where the projected leaf area of *Plantago major* plants grown under different [CO_2_] levels was assessed non-destructively through imaging, followed by destructive measurement of total leaf area and shoot biomass (see Material & Methods).

The first step in establishing a calibration curve involves plotting the variable of interest against the measured variable. We illustrate this process with a graph that depicts the relationship between total leaf area (TLA) and projected leaf area (PLA). The graph demonstrates that for small plants (< 30 cm^2^ in this case, for plants up to 4 weeks old), TLA and PLA exhibit largely similar values. However, in larger plants, TLA increases at a faster rate than PLA, as newly-grown leaves will inevitably overlap partially or even fully with older leaves. In the experiment presented here, the TLA at the final harvest was approximately 2.3 times larger than the corresponding PLA, with no clear difference between plants of the two treatments.

The second step in constructing the calibration curve involves computing a regression line. Taken over both treatments, a linear regression yielded highly significant results (P < 0.001). Based on the calculated r^2^, we found that variation in PLA accounted for 92% of the variation in TLA. While this initial outcome may appear very satisfactory, further examination showed that the regression line *underestimated* TLA at very small and high TLA values, while *overestimating* TLA at intermediate PLA values. Given the gradual increase in leaf overlap with plant size, a curved relationship appears to be a more appropriate model. Subsequent analysis with a second-order polynomial confirmed the high significance (P<0.001) of the quadratic term, resulting in a slightly improved r^2^ (**Table 2**).

**Table 2:** Characterization of different calibration curves for estimating total leaf area (TLA) from projected leaf area (PLA).

	lin	qua	Log(lin)	Log(qua)
P-value for a	***	ns	***	***
P-value for b	***	***	***	***
P-value for c	–	***	–	***
Adj. r^2^	0.920	0.926	0.986	0.989
Df for the error term	162	161	162	161
RMSE (cm^2^)	53.9	51.6	29.9 †	27.3 †
MdAPE (%)	38	25	17 †	12 †

Adj. r^2^: adjusted r^2^; df: degrees of freedom; RMSE: Root Mean Square Error; MdAPE: the Median values of the Absolute Percentage Error. Significance levels: ***, P < 0.001. Equations are of the form y = a + bx for a linear polynomial (lin) or y = a + bx + cx^2^ for a quadratic polynomial (qua). The last two columns are for x and y data that were log_10_-transformed, with the fields marked by a † calculated after back-transformation to the original scale.

Although many users are satisfied with the aforementioned correlative approach and the high r^2^ values (e.g. Nagel et al., 2012; Vadez et al., 2015; Banerjee et al., 2020), certain aspects warrant further inspection. For instance, the growth of young plants often follows an exponential pattern, characterized by smaller absolute size increases in small plants, and larger increases as plants grow bigger. As a consequence, in the experiment we are discussing with weekly harvests, the first half of the calibration curve is determined by 82% of the observations, while the remaining 18% contribute to the second half. To achieve a more balanced distribution, we could log-transform both PLA and TLA. In our experiment, this log-transformation resulted in the first half of the curve containing 32% of the data, with the remaining 68% in the second half (**Figure 6B**). Although the distribution is still not perfectly equal, it improved considerably compared to the non-transformed dataset. Performing a linear regression on the log-transformed data yielded a highly significant fit, with an r^2^ of 0.986. However, it is important to acknowledge the biological phenomenon of overlapping leaves. Incorporating a quadratic term into the equation further improved the fit, resulting in an r^2^ value of 0.989. Clearly, it pays to analyze which function is most appropriate, and whether log-transformation of the data and/or non-linear fits can provide a more robust basis for the calibration curve than a standard linear regression on non-transformed data.

### 3.11 How accurate is the calibration curve?

The coefficient of determination (r^2^) is a convenient parameter to describe the goodness of fit of a statistical relationship between variables, and because it (generally) scales between 0 and 1, it allows easy comparisons across various experiments (Chicco et al., 2021). The calibration curves discussed in the previous section all exhibit relatively high r^2^ values (**Table 2**), but does this r^2^ truly represent the desired accuracy? Different expressions of plant size, such as leaf number, leaf area, and total biomass, are generally well correlated. How well the calibration curve works depends partly on the appropriateness of the chosen proxy trait. For instance: a top-view picture might provide more information for a rosette plant, while side views or views from different angles could be more informative for species with a single stem. Furthermore, it is important to note that r^2^ provides information on the total variation in the y-variable that can be explained by the total variation in the x-variable. Hence, in monotonically increasing relationships like the one depicted in **Figure 6**, the larger the span in size in both x and y, the higher the r^2^ will be. If we restrict the calculation to plants with a PLA >100 cm^2^ instead of considering all data, the r^2^ value decreases from 0.92 to 0.76. Consequently, a high r^2^ for a calibration curve like the one shown in **Figure 6**, indicates that we are able to effectively distinguish between small and large plants. However, if a researcher’s prime interest lies in understanding the variation in final size across genotypes, relying solely on the r^2^ of the full calibration curve may provide a somewhat misleading sense of accuracy.

An alternative measure to assess the goodness of fit is the root mean square error (RMSE), which quantifies the average distance in the y-direction between the observed data points and the fitted line. It gives more weight to points that are further away from the line, compared to those that are closer (Hodson, 2022). The advantage of RMSE is that, all else being equal, it is not influenced by the total variation range in x and y, as is the case with r^2^. Additionally, RMSE provides an absolute error in the units of the Y-axis. If the residuals follow a normal distribution, it informs the researcher that there is a 68% probability that the estimated total leaf area (TLA) deviates by less than the RMSE from the true TLA. However, RMSE may not be suitable for calibration curves that cover a wider range of plant sizes, as the error is not equal for plants of all sizes. For instance, an RMSE of 5 cm^2^ may represent a minor deviation for a plant of 1000 cm^2^, but a huge variation for a plant with a leaf area of 1 cm^2^.

**Figure 7:**
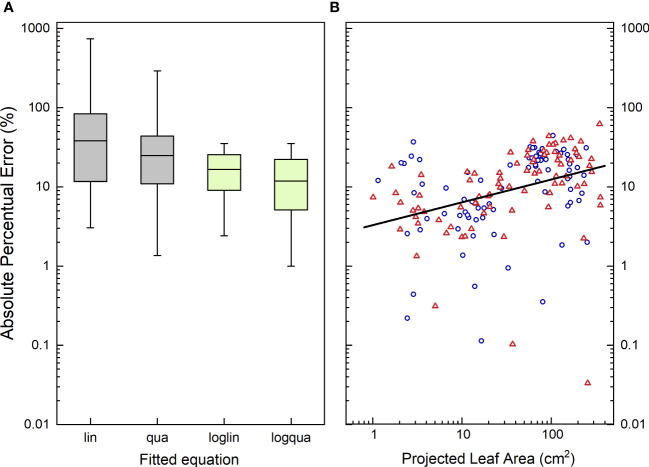
**(A)** Boxplot characterizing the distribution of the Absolute Percentage Error (APE) in the estimate of Total Leaf Area (TLA) from the measurements of Projected Leaf Area (PLA), using four different calibration curves. **(B)** Absolute Percentage Error in the estimate of Total Leaf Area using a log-quadratic calibration curve plotted against Projected Leaf Area. In **(A)**, boxplots indicate the 5^th^, 25^th^, 50^th^, 75^th^ and 95^th^ percentile of the APE values, taken over all plants and treatments. lin, linear regression; qua, quadratic regression; loglin, linear regression through the log_10_-transormed value of TLA and PLA; logqua, quadratic regression through the log_10_-transformed values. In **(B)**, blue circles represent plants grown at low [CO_2_] (350 µL L^-1^), and orange triangles represent high [CO_2_]-grown plants (700 µL L^-1^). The regression line passes through all points, and is significantly (P<0.001) different from zero, with an adjusted r^2^ of 0.15.

To assess the accuracy of the estimates, we calculated for each plant the absolute percentage error. This involved determining the absolute difference between the actual TLA, and the TLA value estimated from the calibration curve, normalized to the actual TLA measured. These values ranged from nearly 0%, indicating a highly accurate estimate, to over 1000% in the specific case of very young plants where TLA was fitted with a straight line across all plant sizes (**Figure 7A**). In the last case, the actual TLA value was 1 cm^2^, whereas the estimated TLA value was calculated to be -10 cm^2^. This illustrates that even an r^2^ value exceeding 0.90 does not necessarily guarantee accurate estimates for every individual plant. The median absolute percentage error (MdAPE) serves as a useful summary descriptor for non-normally distributed data. For the linear calibration curve, MdAPE was approximately 37% (**Table 2**; **Figure 7A**), indicating that the accuracy fell short of our expectations. However, when utilizing a quadratic fit with log-log transformed values, the MdAPE decreased to 11%, signifying a substantial improvement in accuracy. Additionally, in the quadratic fit, MdAPE values were lower for smaller compared to larger ones (**Figure 7B**), which is logical given the increased leaf overlap in larger plants.

The mean absolute percentage error (MAPE) is increasingly utilized in the field of high-throughput plant phenotyping (e.g. Paproki et al., 2012; Paulus, 2019; Rossi et al., 2022). However, given the log-normal distribution of these data, we advocate for the use of the median absolute percentage error (MdAPE) as a more informative measure of the general accuracy. By employing the MdAPE, we aim to capture a representative summary of the actual accuracy, rather than relying solely on the r^2^ of a calibration curve.

### 3.12 How many calibration curves are required?

**Figure 8:**
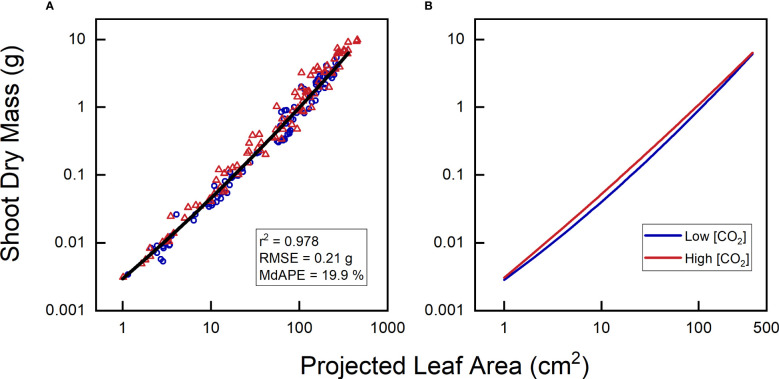
Relationship between projected leaf area and shoot dry mass of *Plantago major*, grown at low {CO_2_] (350 µL L^-1^) and high [CO_2_] (700 µL L^-1^). **(A)** Combined data for plants of both treatments; **(B)** calibration curves separately calculated for plants from each [CO_2_]. All curves exhibited highly significant linear and quadratic components (P < 0.001) and r^2^ > 0.978.

So far, we have considered a common calibration curve for both low and high CO_2_ plants. For the relationship between projected leaf area (PLA) and total leaf area (TLA), this approach may seem reasonable as long as the treatment does not influence leaf angle or any other aspect of leaf display. However, what would happen if we aim to use PLA to estimate shoot biomass (**Figure 8A**)? The relationship between leaf dry mass and leaf area is known to shift, as plants exposed to elevated CO_2_ almost invariably exhibit higher leaf mass per area (LMA; Poorter et al., 2022). Using quadratic polynomials on log-transformed PLA and shoot dry mass, we indeed found different curves (**Figure 8B**). They indicated that for a given PLA, elevated CO_2_ plants were 20-30% heavier, although not for the smallest or largest plants. These findings align with the LMA data, which also showed LMA averages to be 20-30% heavier, except for the first and last harvest (cf. Figure 8B in Poorter et al., 1988). However, statistically, this did not show up as a significant effect, neither for CO_2_ as a main factor nor for the interaction of CO_2_ with the linear and quadratic components of PLA.

Having different calibration curves for different treatments, or maybe for different genotypes, can be quite inconvenient, as it requires more manual harvesting, partially nullifying the intended savings in human effort. What could we do about that? A well-cited method paper by Golzarian et al. (2011) discussed the phenotyping aspects of calibration curves, using the example of an experiment where plants were exposed to salinity or to control conditions. The study found that the two calibration curves were significantly different, with salt-stressed plants exhibiting higher biomass estimates for a given number of pixels compared to control plants. That would fit with the general understanding that salt stress increases leaf mass per area (LMA; Poorter et al., 2009; Kamanga et al., 2023). Golzarian et al. (2011), however, reasoned that salt-stressed plants were smaller, and considered them to be of ‘younger’ age. By adding the factor age to the equation, a single calibration function could be achieved for plants of both treatments. However, this approach mixes plant ontogeny with the direct effects of salinity, and is likely not broadly valid, especially when multiple treatments with varying salinity concentrations would be involved. Consequently, if the treatment of interest affects LMA, leaf angle, or other relevant morphological parameters, different calibration curves for different treatments may indeed be unavoidable.

Another important question to consider is the validity of a calibration curve that has been developed for a particular species, and whether it can be applied to other experiments involving the same species. In growth chambers, where light and temperature stay fixed to the same level, the transferability of a calibration curve seems more likely compared to glasshouses or field settings, where seasonal variation in light and temperature may strongly impact both LMA and stem thickness (and consequently stem mass per projected area). To use HTPP systems more effectively, calibration curves deserve more attention than they got so far. As is custom in many other laboratory methods, it might be good to regularly validate the measuring pipeline with a couple of reference samples that are measured using destructive methods.

### 3.13 How many replicates per genotype or treatment?

Plants grown singly in pots may show quite some variability in plant mass or other traits, which can negatively impact the statistical power to detect differences between genotypes or treatments (Poorter and Garnier, 1996). When planning the size of an HTPP system, it is important to consider not only the number of species or genotypes to be tested, but also the number of replicates per genotype that will be required. Genome-wide association studies (GWAS) or Quantitative Trait Loci (QTL) experiments often benefit more from including additional genotypes rather than increasing the number of replicates per genotype (Zou et al., 2006). If treatments are compared across many genotypes, the large number of plants grown in HTPP systems will provide sufficient statistical power for general conclusions. However, when researchers are also interested in testing specific differences between individual genotypes, the number of replicates becomes more critical. This is particularly true when a calibration curve is used to estimate the values of the trait of interest. Calibration curves with low r^2^ and high MdAPE introduce additional variability on top of the inherent variation that will already be present among plants. In the case of the CO_2_ experiment, a t-test conducted at the final harvest revealed that the actual shoot dry mass for the two treatments was only marginally significant (P = 0.10). However, the difference was far further from significance when the shoot dry mass estimates based on PLA values were used (P = 0.24). In situations where HTPP system users are interested in specific contrasts, the utilization of calibration curves implies that they may need to include more replicates than in traditional experiments to achieve the same level of statistical power.

### 3.14 How often do you need to repeat an experiment?

Despite the robotized and computerized systems, HTPP experiments often push limits and compare large amounts of genotypes in a standardized manner. In as far as these experiments have a background in (eco)physiological approaches, with single plants growing in pots under controlled conditions, data from a single experiment is often considered sufficient for publication. However, in agriculture it is generally regarded as the gold standard to repeat an experiment in multiple years or locations, before any importance is attached to the results.

Possibly, HTPP in controlled conditions can be seen as the initial, important step in a two-phase approach. During this first step, a wide range of genotypes or species can be tested, either in their own right or in combination with specific treatment factors. Without being overly concerned about genotypic effects on calibration curves (as mentioned in point 12), this step can be used to identify the worst-performing and best-performing genotypes, simply based on green pixel counts or similar proxy traits (‘forward phenomics’ *sensu* Mir et al., 2019; cf. Merlot et al., 2002). The most extreme and interesting genotypes, for example those carrying contrasting alleles for important QTLs can then be further investigated in a targeted experiment. This subsequent phase would involve non-destructive phenotyping complemented by more labor-intensive physiological analyses such as gas exchange and chemical characterization on the one hand, and destructive harvesting of both shoot and root biomass on the other hand (Poudyal et al., 2018). Such a two-step approach would also be helpful in screening a wide range of germplasm for contrasting genotypes.

## 4 Conclusions and outlook

In this paper, we discussed a number of issues relevant to consider during the design and implementation phases of high-throughput plant phenotyping (HTPP) systems. A crucial aspect of an HTPP platform is its alignment with the specific research questions of interest and its careful design, both from the hardware and the workflow perspective. Different treatments applied to the shoot environment (such as light, CO_2_, temperature) are often more complicated to implement, as this requires various growth environments all integrated into one HTPP system, or replicated HTPP systems, which is feasible but expensive. Treatments that can be applied to separate pots in the same location (such as drought, nutrients, salinity) are relatively easier to implement and amenable to computerized control. Additionally, efforts must be made to effectively address unforeseen problems and errors.

Regardless of the treatment approach, it is important to acknowledge that investment costs and maintenance requirements for most phenotyping systems are substantial. This is in part because the systems build now are often highly customized. If over time researchers will settle for more standardized systems and sensors, platform costs and time spend on complications will hopefully decrease.

Obtaining meaningful information from HTPP experiments requires to carefully consider the selection of meaningful proxy traits that enable us to answer the research questions at hand. Attention should also be paid to well-designed and regularly validated calibration curves, if necessary. An alternative strategy is to use HTPP systems as a good opportunity for prescreening. Such a prescreening would then be followed by an experiment focusing on a limited number of the most interesting genotypes. It allows to measure not only the proxy variables easily acquired by the phenotyping system (‘soft’ traits), but also the physiological ‘hard-to-get’ traits that provide valuable insights and a more comprehensive understanding of observed differences in plant performance.

## Data availability statement

The original contributions presented in the study are included in the article/**Supplementary Material**. Further inquiries can be directed to the corresponding author.

## Author contributions

HP and PvG initiated the idea, HP, OV and AWK collected data/images. HP wrote the ms. All authors contributed their experiences and viewpoints on phenotyping and commented on the ms. All authors contributed to the article and approved the submitted version.
